# Increased incidence of hypotension in elderly patients who underwent emergency airway management: an analysis of a multi-centre prospective observational study

**DOI:** 10.1186/1865-1380-6-12

**Published:** 2013-04-24

**Authors:** Kohei Hasegawa, Yusuke Hagiwara, Taichi Imamura, Takuyo Chiba, Hiroko Watase, Calvin A Brown, David FM Brown

**Affiliations:** 1 Departments of Emergency Medicine, Massachusetts General Hospital, 326 Cambridge Street, Suite 410, Boston, MA, 02114, USA; 2 Department of Pediatric Emergency and Critical Care Medicine, Tokyo Metropolitan Children’s Medical Center, Fuchu, Japan; 3 Department of Emergency Medicine, Shonan Kamakura General Hospital, Kamakura, Japan; 4 Department of Emergency Medicine, Obama Municipal Hospital, Obama, Japan; 5 Department of Public Health and Preventive Medicine, Oregon Health and Science University, Portland, USA; 6 Departments of Emergency Medicine, Brigham and Women’s Hospital, Boston, USA; 7 Departments of Emergency Medicine, Massachusetts General Hospital, Boston, USA

**Keywords:** Airway management, Emergency department, Elderly, Hypotension, Adverse events

## Abstract

**Background:**

Although the number of elderly increases disproportionately throughout the industrialised nations and intubation-related cardiovascular compromise is associated with hospital mortality, no emergency medicine literature has reported the direction and magnitude of effect of advanced age on post-intubation hypotension. We seek to determine whether advanced age is associated with an increased rate of hypotension at airway management in emergency departments (EDs).

**Methods:**

We conducted an analysis of a multi-centre prospective observational study of 13 Japanese EDs from April 2010 to March 2012. Inclusion criteria were all adult non-cardiac-arrest patients who underwent emergency intubation. We excluded patients in whom airway management was performed for shock or status asthmaticus as the principal indication. Patients were divided into two groups defined *a priori*: age ≥ 65 years old (elderly group) and age < 65 years old (younger group). The primary outcome measure was post-intubation hypotension in the ED.

**Results:**

During the 24-month period, 4,043 subjects required emergency airway management at 13 EDs. Among these, the database recorded 3,872 intubations (capture rate 96%). Of 1,903 eligible patients, 975 patients were age ≥ 65 years (51%) and 928 patients were age < 65 years (49%). The elderly group had a significantly higher rate of post-intubation hypotension compared with the younger group [3% vs. 1%; unadjusted OR 2.7 (95% CI, 1.3–5.6); *P* = 0.005]. In a model controlling for potential confounders (sex, principal indication, method, medication used to intubate, multiple intubation attempts), advanced age had an adjusted OR for post-intubation hypotension of 2.6 (95% CI, 1.3–5.6; *P* = 0.01).

**Conclusions:**

In this large multi-centre study of ED patients who underwent emergent airway management, we found that elderly patients have a significantly higher risk of post-intubation hypotension. These data provide implications for the education and practice of ED airway management that may lead to better clinical outcomes and improved patient safety.

## Background

People aged 65 and older represent approximately 13% of the US population, 40 million in 2010 [1]. US Census data estimate that approximately 20% of Americans will be over age 65 by the year 2030 [2]. Similar trends are apparent throughout the developed world [3]. The disproportionate increase in the number of elderly has been accompanied by an increase in the use of health care, including the emergency department (ED), by patients with comorbid conditions, such as congestive heart failure and coronary artery disease [4].

Many elderly patients with comorbid disease require ED airway management during the course of their illness. In addition to the effect of comorbidities, aging itself brings a number of physiologic changes, such as diminished cardiovascular reserve [5]. Previous studies highlighted that cardiovascular compromise such as post-intubation hypotension in the ED setting is associated with organ dysfunction, prolonged intensive care stay and hospital mortality [6-8]. Although there are several reports that medications used for induction cause more pronounced cardiovascular compromise in elderly patients than in younger patients, most of them are perioperative studies within single institutions [9-13]. Despite its clinical relevance, no emergency medicine literature has reported the direction and magnitude of effect of advanced age on intubation-related cardiovascular events.

Using a robust multi-centre registry of emergency airway management, we aimed to determine whether patients aged 65 years or older have a higher incidence of post-intubation hypotension in the ED.

## Methods

### Study design and setting

This study was an analysis of the Japanese Emergency Airway Network (JEAN) Registry, a prospective observational multi-centre data registry, with all data collection planned *a priori*. The study setting, methods of measurement and measured variables have been reported previously [14,15]. In summary, JEAN was a consortium of 13 academic and community medical centres from different geographic regions across Japan. All 13 EDs were staffed by emergency attending physicians, and 12 had affiliations with emergency medicine residency training programs. The participating institutions were certified as level I (*n* = 10) or level II equivalent (*n* = 3) trauma centres. They had a median ED census of 30,000 patient visits per year (range, 9,000 to 67,000). The study was approved by the Institutional Review Board of each participating centre with waiver of informed consent prior to data collection and performed in accordance with the Helsinki Declaration.

### Selection of participants

All adult patients aged 18 years or older who underwent emergency tracheal intubation at a participating ED during a 24-month period (April 2010 to March 2012) were included in the study. We excluded those patients in whom airway management was performed for cardiac arrest, shock (systolic blood pressure < 90 mmHg) or status asthmaticus as the principal indication.

### Data collection

Case ascertainment was passive, relying on self-reporting by intubators on duty in the ED. After each intubation, the intubator completed a standardised form that included the patient’s age, sex, indication for intubation, methods of intubation, devices and all medications used to facilitate intubation, operator level of training and specialty, number of attempts, success or failure, and intubation-related adverse events. We monitored compliance continuously by reviewing professional billing codes, cross-referencing our findings with the intubation data forms. When a patient underwent an intubation but for whom we had received no data collection form, the intubator was interviewed by one of the investigators to fill out the data.

### Outcome measures

During the development phase, key definitions were agreed on. “Adverse events” were *a priori* defined as intubation-associated events with two categories: hypotension and other adverse events. Hypotension was defined as any recorded systolic blood pressure < 90 mmHg after airway management in EDs [16]. Other adverse events included dysrhythmia, cardiac arrest, death, oesophageal intubation with delayed recognition, dental or lip trauma, mainstem bronchial intubation, regurgitation, hypoxemia and airway trauma. Cardiac arrest includes asystole, bradycardia or dysrhythmia with non-measurable blood pressure and cardiopulmonary resuscitation during or after intubation. Oesophageal intubation was defined as misplacement of the tracheal tube in the upper oesophagus or hypopharynx with a lapse of time and clinical deterioration, such as hypoxemia, before the removal of the misplaced tube [16-18]. Regurgitation was defined as gastric contents that required suction removal during laryngoscopy in a previously clear airway. Hypoxemia was defined as pulse oximetry saturation < 90%.

An intubation “attempt” was defined as a single insertion of the laryngoscope (or other device) past the teeth. An attempt was successful if it resulted in a tracheal tube being placed through the vocal cords with confirmation by a quantitative or colorimetric end-tidal carbon dioxide monitor [16]. Multiple intubation attempts were defined as three or more intubation attempts [19].

### Statistical analysis

We analysed the compiled data with simple descriptive statistics. Continuous data are presented as means and standard deviations (SD) or medians and interquartile ranges (IQRs) as appropriate based on distribution of the data; categorical data are reported as proportions and 95% confidence intervals (CIs). For the purposes of this analysis, the cohort was divided into two groups defined *a priori* based on the patient’s age: patients aged 65 years or older (elderly group) and patients aged less than 65 years (younger group). These classifications were defined in a written protocol prior to querying the database or analysing any data.

The primary outcome measure was a post-intubation hypotension. The occurrence of the primary outcome was compared between the groups using the chi-square test for the difference in proportions. The secondary outcome measure was the occurrence of any adverse event. Odds ratios (OR) were calculated to determine independent predictors of adverse events. Given the dichotomous outcome, we performed multivariable logistic regression modelling, adjusting for important patient and ED factors that have been shown to predict adverse events [16,19-23]. These factors included sex, principal indication for intubation, initial method of intubation, sedatives and multiple intubation attempts. In the regression model, the predictive effects on the rate of adverse events were assessed for age ≥ 65 years. This elderly group was coded as a contrast variable against age < 65 years. We tested for effect modification between rapid sequence intubation and sedatives by including their interaction terms in a model with adverse event as the outcome (data not shown). Results did not indicate effect modification and so interaction terms were not included in the final model. The discrimination and calibration of the model was determined by using the c-statistic and Hosmer-Lemeshow test, respectively.

In the sensitivity analyses, we repeated the multivariable analysis, comparing patients aged 80 years or older versus patients aged less than 80 years. We also conducted an analysis modelling the age as an ordinal variable. All ORs are presented with 95% CIs. All *P* values are two-sided, with a *P* value of less than 0.05 considered statistically significant. Data analyses were conducted with SAS statistical software (version 9.3; SAS Institute, Cary, NC).

## Results

### Characteristics of study subjects

**Figure 1:**
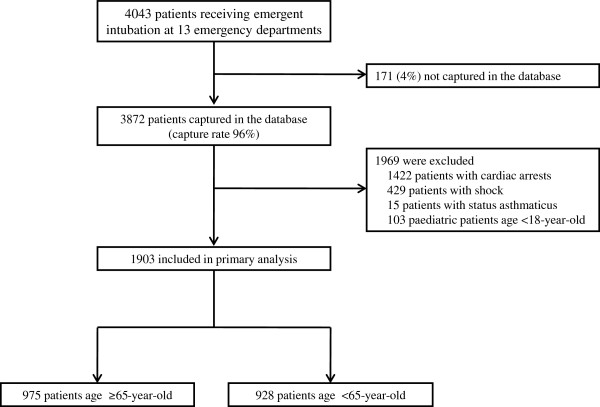
Study flow.

During the 24-month period, there were 4,043 subjects requiring emergency airway management at 13 EDs (Figure 1). Among these, the database recorded 3,872 intubations (capture rate 96%). We excluded 1,866 patients who underwent airway management for cardiac arrest, shock or status asthmaticus and 103 patients who were aged younger than 18 years. The remaining 1,903 patients were analysed in the study.

Baseline characteristics for two groups appear in Table 1. The overall median age was 65 years; 51% were 65 years or older (*n* = 975). Most intubation involved medical emergencies. ED airway management choices are displayed in Table 2. Sedatives, without neuromuscular blockade, were chosen in 682 patients (36%) of the 1,903 intubations; rapid sequence intubation was the initial method chosen in 542 (28%). Benzodiazepine was administered as the sedative in 630 patients (22%), propofol in 447 (23%) and ketamine in 66 (3%). Of the 1,903 patients, 1,648 were successfully intubated within ≤ 2 attempts (87%). Ultimately, intubation was successful in 1,898 patients (99.7%).

**Table 1:** Baseline characteristics of 1,903 patients who underwent emergent airway management, by age group

**Patient characteristics**	**All patients (*n*** **= 1,903)**	**Age ≥ 65 years (*n*** **= 975)**	**Age < 65 years (*n*** **= 928)**
Age, median (IQR), years	65 (50–77)	77 (70–82)	50 (36–58)
Female sex (%)	773 (40)	431 (44)	342 (37)
Primary indication (%)			
Medical encounters	1,603 (84)	840 (86)	763 (82)
Altered mental status	938 (49)	395 (41)	543 (59)
Respiratory failure	515 (27)	358 (37)	157 (17)
Airway obstruction	116 (6)	77 (8)	39 (4)
Other medical	19 (1)	5 (1)	10 (1)
Trauma encounters	300 (16)	135 (14)	165 (18)
Head trauma	182 (10)	79 (8)	103 (11)
Facial/neck trauma	53 (3)	28 (3)	25 (3)
Burn/inhalation	42 (2)	19 (2)	23 (2)
Other trauma	23 (1)	9 (1)	14 (2)

*IQR*, interquartile range. Percentages may not equal 100 due to rounding.

**Table 2:** ED airway management characteristics in 1,903 study patients, by age group

**Management**	**All patients(*n*** **= 1,903)**	**Age ≥ 65 years(*n*** **= 975)**	**Age < 65 years(*n*** **= 928)**
Initial method (%)			
Sedation without paralysis	682 (36)	360 (37)	322 (35)
Oral without medication	546 (29)	298 (31)	248 (27)
Rapid sequence intubation	542 (28)	269 (28)	273 (29)
Other*	133 (7)	48 (5)	85 (9)
Initial device (%)			
Direct laryngoscope	1,810 (95)	933 (96)	877 (95)
Video laryngoscope	44 (2)	17 (2)	27 (3)
Other†	49 (3)	25 (3)	24 (2)
Sedative (%)			
No sedatives	724 (38)	372 (38)	352 (38)
Benzodiazepine	630 (22)	343 (35)	287 (31)
Propofol	447 (23)	205 (21)	242 (26)
Ketamine	66 (3)	42 (4)	24 (3)
Other‡	36 (2)	13 (1)	23 (2)
Paralytic (%)			
No paralytics	1,291 (68)	689 (71)	602 (65)
Rocuronium	402 (21)	177 (18)	225 (24)
Vecuronium	150 (8)	75 (8)	75 (8)
Succinylcholine	60 (3)	34 (3)	26 (3)
Specialty of first intubator (%)			
Transitional year resident§	703 (37)	384 (39)	319 (34)
Emergency medicine resident	648 (34)	298 (31)	350 (39)
Emergency physician	348 (18)	180 (18)	168 (18)
Other specialties¶	202 (11)	113 (12)	89 (10)
Number of intubation attempts, median (IQR)	1(1–2)	1(1–2)	1 (1–2)
> 3 intubation attempts (%)	255 (13)	128 (13)	127 (14)
Ultimate intubation success (%)	5 (< 1)	3 (< 1)	2 (< 1)

*IQR*, interquartile range.

*Defined as oral intubation using paralytics without sedatives, transnasal intubation or cricothyrotomy.

†Defined as oral intubation using a bougie, lighted stylet, laryngeal mask airway and fibroscopy, transnasal intubation or cricothyrotomy.

‡Defined as administration of thiopental, haloperidol or combination with any of the included sedative categories.

§Defined as post-graduate years 1 and 2.

¶Defined as surgery, anaesthesia or paediatrics.

## Main results

Overall, 2% of patients (*n* = 38) met the primary outcome of post-intubation hypotension (Table 3). The rate of hypotension was significantly higher in the elderly group compared with the younger group (3% vs. 1%; unadjusted OR, 2.7; 95% CI 1.3–5.6; *P* = 0.005). After adjustment for potential confounders, advanced age was independently associated with post-intubation hypotension (adjusted OR, 2.6; 95% CI, 1.3–5.6; *P* = 0.01) (Table 4). The c-statistic for the model was 0.80, and the Hosmer-Lemeshow test demonstrated a good fit (*P* = 0.55). In the sensitivity analyses, the adjusted association of increased rate of post-intubation hypotension with advanced age persisted with the use of different definitions for advanced age.

**Table 3:** Unadjusted adverse event rates by age group, by age group

	**All patients**	**Age ≥ 65 years**	**Age < 65 years**	**Unadjusted OR for age ≥ 65 years**	***P *Value**
Adverse events*	**(*n*** **= 1,903)**	**(*n*** **= 975)**	**(*n*** **= 928)**	**(95% CI)**	
Hypotension	38 (2)	28 (3)	10 (1)	2.7 (1.3–5.6)	0.005
Other adverse events					
Dysrhythmia	2 (< 1)	0	2 (< 1)	†N/A	†N/A
Cardiac arrest	6 (< 1)	3 (< 1)	3 (< 1)	1.0 (0.2–4.7)	0.95
Death	6 (< 1)	2 (< 1)	4 (< 1)	0.5 (0.1–2.6)	0.38
Oesophageal intubation with delayed recognition	90 (5)	45 (5)	46 (5)	0.9 (0.6–1.4)	0.82
Dental/lip trauma	68 (4)	29 (3)	39 (4)	0.7 (0.4–1.1)	0.15
Mainstem bronchus intubation	32 (2)	22 (2)	10 (1)	2.1 (1.0–4.5)	0.05
Regurgitation	35 (2)	10 (1)	25 (3)	0.4 (0.2–0.8)	0.007
Hypoxemia	8 (< 1)	4 (< 1)	4 (< 1)	1.0 (0.2–3.8)	0.94
Airway trauma	5 (< 1)	2 (< 1)	3 (< 1)	0.6 (0.1–3.8)	0.61
All groups combined	256 (13)	132 (14)	124 (13)	1.0 (0.8–1.3)	0.91

*CI*, confidence interval; *N/A*, not analysed.

*Patients may have more than one adverse event.

†The odds ratio for this variable was not calculated because there were too few events.

**Table 4:** Multiple logistic regression model with cardiovascular adverse event and any adverse event as the dependent variable

	**Post-intubation hypotension**		**Overall adverse event**	
**Variable**	**OR (95% CI)**	***P *Value**	**OR (95% CI)**	***P *Value**
**Primary exposure**				
Age ≥ 65 years (vs. < 65 years)	2.6 (1.3–5.6)	0.01	1.0 (0.8–1.3)	0.97
Age (categorical variable)*				
Age ≥ 80 years (vs. < 80 years)	2.4 (1.2–4.8)	0.01	0.98 (0.7–1.4)	0.92
Age, decile (ordinal variable: OR per each incremental decile)*	1.3 (1.1–1.7)	0.01	0.98 (0.9–1.1)	0.66
**Covariate**				
Female (vs. male)	1.1 (0.6–2.2)	0.75	1.5 (1.1–1.9)	0.009
Primary indication		0.68		0.88
Medical	1.2 (0.5–3.2)		1.0 (0.7–1.5)	
Trauma	1 [reference]		1 [reference]	
Sedative (%)		0.001		0.14
No sedatives	0.1 (0.01–1.0)		1.2 (0.8–1.7)	
Benzodiazepine	3.6 (1.3–10.0)		1.5 (1.0–2.3)	
Propofol	1 [reference]		1 [reference]	
Ketamine	1.0 (0.1–9.2)		0.9 (0.4–2.2)	
Other†	5.9 (1.0–31.8)		2.1 (0.9–4.9)	
Method		0.58		0.73
RSI	1.2 (0.6–2.5)		1.1 (0.7–1.6)	
Non-RSI‡	1 [reference]		1 [reference]	
Multiple attempts§ (vs. 2 or fewer attempts)	(0.4-2.6)	0.98	4.7 (3.4-6.4)	< 0.001

*OR*, odds ratio; *CI*, confidence interval; *RSI*, rapid sequence intubation.

*Sensitivity analyses.

†Defined as administration of thiopental, haloperidol or combination with any of the included sedative categories.

‡Defined as oral intubation without medication, with sedatives only, or with paralytics only, transnasal intubation and cricothyrotomy.

§Defined as 3 or more intubation attempts.

The overall adverse event rate was 13% (*n* = 256; Table 3). There was no significant difference in the overall adverse event rate between the elderly and younger group (14% vs. 13%; unadjusted OR, 1.0; 95% CI, 0.8–1.3; *P* = 0.91). This association remained non-significant after adjusting for predefined factors using a multivariable regression model (adjusted OR, 1.0; 95% CI, 0.8–1.3; *P* = 0.97; Table 4).

## Discussion

In this large prospective multi-centre study of adult non-cardiac-arrest patients who underwent ED airway management, we found that patients aged 65 years or older had a significantly higher rate of post-intubation hypotension. Furthermore, after adjusting for a predefined set of confounding variables in a multivariable analysis, we found that advanced age was an independent predictor of post-intubation hypotension.

Our findings are difficult to compare with others’ results because this topic is not well addressed in previous research. Reich et al. performed a retrospective analysis involving 4,096 anaesthesia records from a single centre [24]. The author found that age 50 years or older was independently associated with the occurrence of hypotension after general anaesthesia. A previous smaller study from Hong Kong involving 160 ED patients found that midazolam caused more hypotension than etomidate, particularly in elderly patients [13]. Although this study assessed the intubation-related adverse events in the ED setting, the study hypothesis was not explicitly stated and the association between age and adverse events was not significant likely because of type II errors. To our knowledge, this is the first emergency medicine study providing evidence that the incidence of post-intubation hypotension accelerates with advanced age.

Aging affects cardiac function in many ways. Stiffening of large arteries increases after-load, while myocardial stiffening impairs early diastolic filling [25,26]. Conduction abnormalities and bradyarrhythmias are more prevalent in the elderly, potentially contributing to cardiac arrest [27]. Additionally, chronic illness and disability are common in the elderly. Exacerbations of chronic cardiac diseases often exhaust the patient’s limited cardiovascular reserve, leading to hypotension, dysrhythmia and cardiac arrest. Diminished overall cardiovascular reserve in elderly patient results in heightened sensitivity to the negative inotropy and vasodilatory effects of induction agents and other vasoactive drugs. Furthermore, a drop in diastolic pressure and coronary perfusion pressure can have deleterious effects in patients with preexisting coronary artery disease. In elderly patients, the goals of airway management are not only to provide appropriate intubation condition, but also to preserve myocardial and haemodynamic function, control for the effects of pre-existing disease and avoid adverse events such as hypotension or dysrhythmia [28].

Out data establish an association between advanced age and post-intubation hypotension in the ED. In contrast, we cannot evaluate the effect of intubation-related hypotension in elderly patients on long-term mortality as this registry was not designed to measure patient outcomes after ED dispositions. One may surmise that a transient adverse event in the ED setting is a benign or self-limited consequence of airway management [29]. Indeed, hypotension has been described as a physiologic response to intubation caused by multiple mechanisms, such as induction-associated sympatholysis and the effects of positive pressure ventilation [30]. However, intubation-associated hypotension is associated with long-term morbidity and mortality across differing patient populations and clinical settings [24,30-33]. In the setting of exacerbations of chronic cardiopulmonary diseases, frank hypotension induced by intubation may act as a secondary insult that incites or advances hypoperfusion and directly contributes to organ dysfunction in critically ill elderly patients who undergo emergency airway management. Thus, advanced age may represent a high-risk marker of cardiovascular outcomes that warrants an early and organised haemodynamic resuscitation approach.

Our findings have important implications for the practice and training of emergency physicians and provide an opportunity to rethink the approach to airway management in critically ill elderly patients for the promotion of patient safety. Safe and effective emergency airway management, especially for patients with tenuous haemodynamics or poor cardiovascular reserve, requires specialty care provided by residency trained emergency physicians, anaesthesiologists and critical care physicians who can safely combine volume resuscitation, judicious use of vasoconstrictors and appropriate doses of induction agents. Improvements in training may lead to higher success rates as well as lower adverse event rates. Nevertheless, our data do not provide specific evidence that an approach with specific medications or methods would provide better clinical outcomes or fewer cardiovascular adverse events in elderly patients. We hope that this ongoing registry will compile a larger data set to allow for testing of this clinical question.

### Limitations

We acknowledge important limitations in this study. First, passive surveillance introduces the potential of reporting bias. Therefore, underreporting of the number of adverse events is possible even though a previously applied reporting system with structured data forms with uniformed definitions and a high capture rate limit this possibility. Furthermore, assuming that underestimation of the number of adverse events occurred evenly in the both groups, this non-differential misclassification would not have biased our inference. Second, our patients were monitored via noninvasive or invasive blood pressure assessment, and post-intubation haemodynamic changes may have gone undetected because of the intermittent nature of this monitoring. In addition, mean arterial pressure, which might be more relevant to septic patients, was not measured in this study. Another important limitation is that, as with any observational study, the association between advanced age and the increased rate of post-intubation hypotension does not prove causality and might be confounded by unmeasured factors. For instance, potential confounding variables may include underlying cardiovascular risk. We did not have this information. However, we did adjust for primary indication to help account for this possible confounder. Furthermore, we attempted to minimise other confounding factors by adjusting for sex, indication, method of intubation, sedatives and multiple intubation attempts. Finally, we recognise that no patients received etomidate as it has not been approved by the Japanese Ministry of Health, Labour and Welfare. Therefore, these results may not be generalisable to those patients intubated using etomidate. However, our observation is valuable in resource-limited environments such as developing countries where etomidate is unavailable.

## Conclusions

In this large multi-centre cohort of adult ED patients who underwent emergent airway management, we demonstrated that age 65 years or older was an independent predictor of post-intubation hypotension. These data provide implications for the education and practice of ED airway management that may lead to better clinical outcomes and improved patient safety.

## Abbreviations

CI: Confidence interval; ED: Emergency department; IQR: Interquartile range; JEAN: Japanese emergency airway network; OR: Odds ratio; SD: Standard deviation.

## Competing interests

The authors declare that they have no competing interests.

## Authors’ contributions

KH and DFMB conceived the study. KH obtained research funding. KH, YH, HW, TC and CAB supervised the conduct of the trial and data collection. YH, HW and TC managed the data, including quality control. YH and HW provided statistical advice on study design and analysed the data; KH chaired the data oversight committee. KH and TI drafted the manuscript, and all authors contributed substantially to its revision. KH take responsibility for the paper as a whole. All authors read and approved the final manuscript.
